# SMILE body project: protocol for a randomized controlled trial of an online eating disorder prevention program in young women with psychiatric disorders

**DOI:** 10.1007/s40519-026-01832-w

**Published:** 2026-03-11

**Authors:** Caroline Bruun Abild, Eric Stice, Pernille Byrial, Gry Kjaersdam Telléus, Loa Clausen

**Affiliations:** 1 Steno Diabetes Center Aarhus, Aarhus University Hospital, Aarhus, Denmark; 2 Department of Child and Adolescent Psychiatry–Research unit, Aarhus University Hospital Psychiatry, Aarhus, Denmark; 3 Department of Psychiatry and Behavioral Sciences, Stanford University, Stanford, USA; 4 Department of Clinical Medicine, Aarhus University, Aarhus, Denmark; 5 Psychiatry, Aalborg University Hospital, Aalborg, Denmark; 6 Department of Communication and Psychology, Aalborg University, Aarhus, Denmark

**Keywords:** Eating disorder, Prevention, Study protocol, Psychiatric illness, Body dissatisfaction

## Abstract

**Aim:**

Young women with psychiatric illness have an increased susceptibility for eating disorders (ED); however, no prevention initiatives have been tested for this group. The Body Project ED prevention program has produced promising effect on ED symptoms, body satisfaction, and future ED onset; however, no studies have investigated this specific vulnerable group. In SMILE Body Project, we adapted the Body Project manual to a psychiatric population. In this protocol paper, we describe the randomized controlled trial (RCT) to evaluate the efficacy of the SMILE Body Project.

**Methods:**

This is an ongoing RCT including 300 young women between 15 and 30 years with a psychiatric illness and subjective body dissatisfaction, starting May 2025. Participants are randomized to either online Body Project groups or an active control arm based on expressive writing exercises. The primary aim is to test if SMILE Body Project significantly reduces the incidence of ED in women with psychiatric illness after 2 years compared to controls. The secondary aim is to explore changes in ED symptoms, psychiatric symptoms, QoL, and predictors such as psychiatric symptoms and time spent on social media.

**Results:**

We expect that SMILE Body Project will effectively reduce the incidence of ED diagnosis compared to controls. In addition, we anticipate new knowledge on how time spent on social media affects body image in women with psychiatric illness.

**Conclusions:**

If SMILE Body Project proves efficacious, it has potential for implementation at low cost and may contribute to broad prevention of future ED onset among young women with psychiatric illness.

**Clinical Trials. gov Identifier:**

NCT06893627

**Supplementary Information:**

The online version contains supplementary material available at 10.1007/s40519-026-01832-w.

## Introduction

Eating disorders (ED) are characterized by body dissatisfaction, dietary restraint, binge eating, and/or compensatory behaviors, such as self-induced vomiting, excessive exercise, and misuse of laxatives and diuretics, with the main diagnostic categories being anorexia nervosa, bulimia nervosa, and binge eating disorder (DSM-5) [1]. EDs are associated with functional impairment, reduced quality of life (QoL), increased co-morbidity, and mortality [2, 3]. Peak incidences for most ED occur between the ages of 16–25 [4] indicating that ED prevention initiatives should be implemented within this specific age group or earlier [5, 6].

Health care costs for EDs, especially anorexia nervosa, are high, few receive diagnosis and treatment, and among those who do, fewer than 50% produce symptom remission [7, 8]. In addition, individuals with binge eating disorders and bulimia nervosa often go untreated for years or even decades [9, 10]. Thus, there is a need for effective evidence-based prevention programs to reduce future onset of ED in the general population, but especially in high-risk groups.

Individuals with other primary disorders such as psychiatric illness and chronic diseases have increased risk of EDs [11, 12]. The risks are fourfold among those with psychiatric illness [12]. For individuals with psychiatric illness, the increased risk might be related to the elevated distress due to psychiatric symptoms or due to interpersonal problems related to the primary disorder or possible shared genetic factors [13]. One of the most dominant risk factors, and primary drivers of EDs, is body dissatisfaction [14]. Body dissatisfaction has been on the rise among adolescents in recent decades [15].

The Body Project is a dissonance-based intervention that helps participants understand how society and social media (SoMe) affect our perception of body and appearances. It is currently the most studied ED prevention program, reducing ED symptoms and future onset of ED by up to 77% (relative risk) over 2 years compared to the control group [16].

The Body Project consists of four 1 h group sessions led by trained facilitators and has primarily been tested in females with body image concerns [17]. The program was developed at Stanford University and the University of Texas at Austin, USA, by Professor Eric Stice [18]. During and between sessions, participants are encouraged to engage in verbal, written, and behavioral exercises in which they collectively critique the thin beauty ideal. These activities lead to a reduction in thin-ideal endorsement by inducing cognitive dissonance, which motivates participants to adjust their beliefs and attitudes to align with the perspectives expressed in the sessions [19]. The reduction in pursuit of the thin ideal results in subsequent decreases in body dissatisfaction, unhealthy dietary restriction, and negative affect, which reduces risk for future ED onset.

Body Project produces significantly greater reductions in thin idealization, body dissatisfaction, dieting, negative affect, and ED symptoms compared to control groups among 15 + year females at post-test and at 2–3-year follow-up [20].

The Body Project is most effective in reducing the onset of new ED when facilitated or co-facilitated by peers. A recent meta-analysis found that the peer-led in-person Body Project intervention reduced the future onset of eating disorders by 58% over a period of 4 years [17]. The Body Project has not reduced future onset of ED when facilitated solely by clinicians [21]. Proposedly, health promotion interventions are perceived as more credible if delivered by individuals who are similar to group participants [22]. Previous studies have mostly been in-person, but recent studies point toward a similar efficacy when delivered virtually [16, 23].

Considering the significant physical and psychological impacts of EDs, it is imperative that research focus on effective and implementable ED prevention. With four online sessions, the Body Project is an easily accessible, scalable, and cost-effective approach to reduce the future incidence of ED and improve overall well-being among vulnerable young women.

## Objectives of the SMILE trial

The present study aims to test the efficacy of the Body Project as a virtually delivered peer-led intervention for women with psychiatric illness and body dissatisfaction.

The primary aim of this study is to reduce the incidence of ED, measured by a clinical diagnostic interview after 2-year follow-up.

Secondary objectives are to examine whether the Body Project intervention leads to (1) reductions in eating disorder symptoms, body dissatisfaction, and thin-ideal internalization, and (2) improvements in QoL, loneliness, and overall psychiatric symptom burden across follow-up assessments.

The Body Project manual has been adapted to include elements relevant for individuals with psychiatric illness, with an increased emphasis on SoMe-related content.

After baseline assessment, participants are randomized to either:

(1) Body Project intervention, or (2) Active expressive writing control condition.

We propose the following hypothesis:

I. The Body Project intervention will be effective in reducing incidence of ED (primary outcome) over a 2-year period compared to controls.
II. The Body Project intervention will significantly reduce ED symptoms, body dissatisfaction, and thin idealization at all follow-up assessments.
III. The Body Project will be effective in reducing total psychiatric symptom load, loneliness and QoL at all follow-ups compared to active controls.
IV. Baseline psychiatric symptoms, SoMe use, and body dissatisfaction predict future increases in ED symptoms over a 2-year period.

## Methods

### Research design

A randomized controlled methodology was applied in this study.

### Participants

**Fig. 1:**
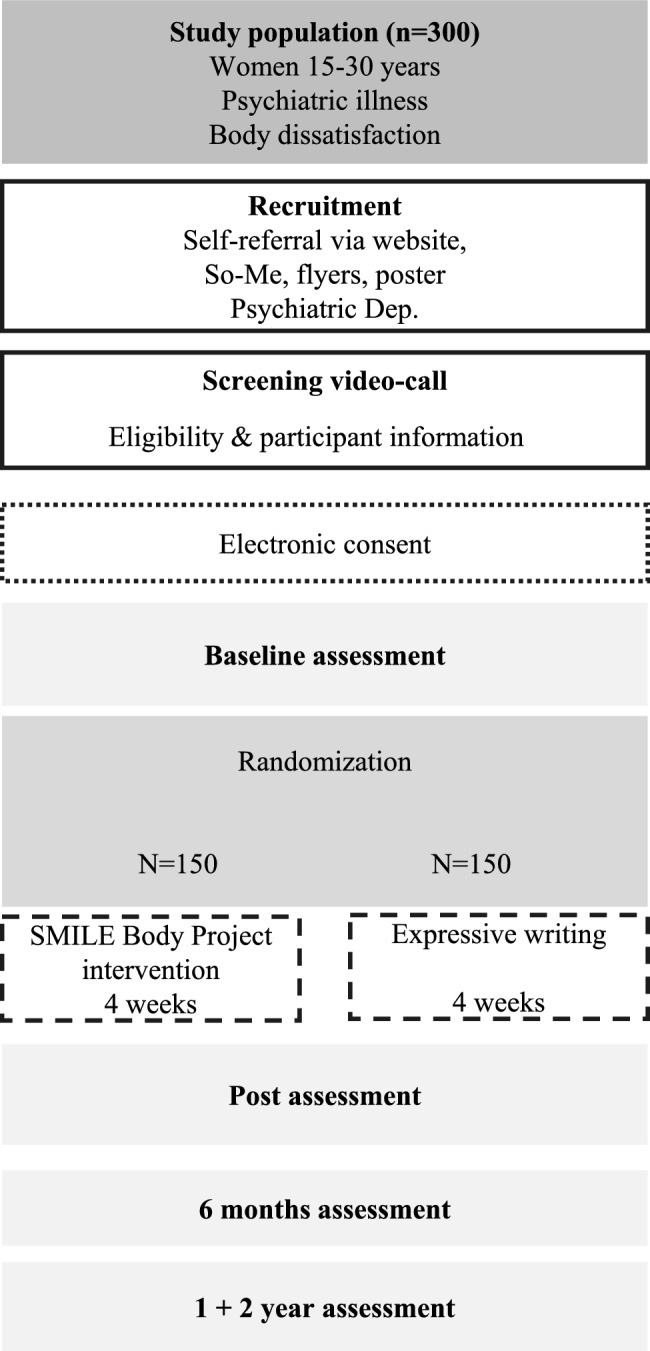
Participant flowchart of the body project SMILE trial

We aim to recruit 300, 15–30-year-old, women with a psychiatric diagnosis, randomized 1:1 to the Body Project intervention or an active control condition. Participants are allocated using block randomization with varying block sizes and random permutations within each block to ensure allocation concealment and balance across study arms. All participants receive usual care for their psychiatric condition. A participant flowchart is illustrated in Fig. 1.

### Recruitment

All participants are self-referrals. Information on the project is available as leaflets, either in print or in SoMe (Instagram and TikTok). A specific research website (www.bodyproject.dk) has been established and contains participant information and contact information. The specific wording in the recruitment “Do you want to beat your body dissatisfaction?” will aid in the natural selection of women at high risk. In addition, a registration form for expression of interest is available. Psychiatric Departments throughout Denmark and educational organizations (upper secondary schools, high schools, vocational educations) in Central Denmark Region and Northern Denmark Region can provide information about the project and hand out the information leaflets. The participant information and information sent to parents to participants < 18 years of age are similar, except for added parental guidance on how best to support their adolescents.

### Inclusion criteria

The following inclusion criteria are utilized: (1) women; (2) 15–30 years; (3) at least one medically verified psychiatric diagnosis diagnosed (e.g., mood disorders, anxiety disorders, ADHD, and personality disorders); and (4) self-reported body dissatisfaction.

Participants may have more than one psychiatric diagnosis, and all psychiatric diagnoses will be recorded at baseline using ICD-10 codes.

### Exclusion criteria

Current ED diagnosis of anorexia nervosa, bulimia nervosa, or binge eating disorder according to DSM-5 criteria or severe subtypes of these corresponding to other specified ED (e.g., clearly self-induced low weight below the 5th percentile for persons below 18 years of age or BMI 17½ in persons 18 + years but without disturbed body perception, vomiting twice a week or more for 3 months but with objective binge being absent or less frequent) as evaluated via the Eating Disorder Diagnostic Interview or history of an ED within the past 12 months. Prodromal cases are included.

Acute psychiatric instability or psychosis or suicide attempt within the last 12 months, or inability to cognitively understand the project or work in the online group setting.

Both inclusion and exclusion criteria are presented in Table 1.

**Table 1:** Inclusion and exclusion criteria

Inclusion criteria	Exclusion criteria
Female participants	Current ED diagnosis or clinically significant ED symptoms (DSM-5, via Eating Disorder Diagnostic Interview) or history of an ED within the past 12 months
Aged 15–25 years	Acute psychiatric instability, psychosis, or suicide attempt within past 12 months
Medically verified psychiatric diagnosis (e.g., mood disorders, anxiety, ADHD, and personality disorders)	Cognitive inability to engage in the project
Self-reported body dissatisfaction	Severe substance abuse impairing participation

### The SMILE body project intervention

Minor cultural or disease-specific adaptations to the general Body Project manual have been made, in collaboration with both Stice, including participants’ perspectives. The SMILE Body Project will consist of 4 sessions. An overview of content is provided in Table 2. Sessions are held via a secure online platform, uniquely applicable to invited participants. Sessions are recorded as this will enable supervision and fidelity assurance to ensure compliance with the manual. Short individual follow-up contacts via phone will be made if participants miss a session.

**Table 2:** Overview of SMILE body project sessions

Session	Content	Home exercises
1	• Discussions, definition and origins of the thin ideal, and costs associated with pursuing the thin ideal	Write a **Letter to a younger girl** struggling with body image concerns including costs associated with pursuing the thin ideal **Mirror exercise,** stand in front of a mirror and look at yourself and write down 10 positive qualities. This includes physical, emotional, intellectual, and social qualities
2	• Present and discuss homework from session 1 • Role plays to elicit verbal statements against the thin ideal	**Talk to two people** and ask them to discuss the main costs of pursuing the thin ideal Generate a **Top-10 list** of things girls/women can do to resist the appearance ideal
3	• Present and discuss homework from session 2 • Role plays and discussions how to resist the thin ideal, how to challenge personal body-related concerns, and how to respond to future pressures to be thin • Discussion on the influence of social media on body image perception	As a **Behavioral Challenge**, do two things that you currently do not do because of body image concerns to increase your confidence Please choose two behaviors from your Top-10 list to do between sessions as a **Body Activism** exercise Choose at least one way to challenge appearance ideals and promote a healthier approach to **social media** Write a letter or create a list of advice to your y**ounger self**, using what you’ve learned in the group to show how to prevent body image concerns and build a positive self-image
4	• Present and discuss homework from session 3 • Discuss how the ways in which we talk about our bodies may promote the thin ideal • Have participants come up with more positive alternative ways of talking about their bodies and encouraging participants to continue to challenge their body image issues in the future	As a **self-affirmation** exercise, choose three ideas to keep challenging your body image concerns and work toward developing a positive body image

Each group will consist of 6–8 group members as in previous studies [16, 23, 24]. The groups are led by two peers. The peers are trained young adults, preferably with personal experience of psychiatric illness, i.e., students within the area of health care (medicine, dietetics, psychology, and nursing).

All peers have received extensive and continuous training and supervision. The training will be made available as online sessions based on the manuscript and in collaboration with Eric Stice. Fidelity assurance and continuous monitoring and evaluation of sessions will be performed during bimonthly supervision. Previous fidelity testing has shown high inter-rater agreement for the original Body Project [25].

### Active control condition

The expressive writing (EW) serves as the active control condition and will consist of written instructions sent to participants weekly via text message over 4 weeks, asking them to write about their thoughts, emotions, images, and other content related to their body. They are encouraged to sit undisturbed for 40 min (no more, no less) and write only to themselves. If, after a while, they do not know what to write, they are encouraged to repeat what they have already written. This version of EW was used as earlier trials have found them equally credible to the Body Project [16].

The output is solely for the participants themselves and will not be read by others.

## Measures

Applying measurements from existing Body Project literature will allow for comparison to effects reported in previous trials, which will facilitate interpretation of our findings. All questionnaires will be set up in REDCap. Questionnaires have been translated and back-translated according to guidelines [27].

### Primary outcome measures

*ED diagnosis*: The semi-structured Eating Disorder Diagnostic Interview (EDDI) assesses ED symptoms, including binge eating and purging, and will be used to determine anorexia nervosa, bulimia nervosa, binge eating disorder, and purging disorder [28]. The interview will be performed by video/telephone by an experienced clinician/researcher.

### Secondary outcome measures

ED risk factors and symptoms:

- *Thin idealization*: Ideal-Body Stereotype Scale–Revised to assess pursuit of the thin ideal [29].
- *Body dissatisfaction*: Body Dissatisfaction Scale to assess dissatisfaction with various body parts [21, 30].
- *Overevaluation of appearance, weight and shape* will be measured by selected items from the Eating Disorder Examination Questionnaire Eating behavior (EDE-Q) items 22, 23
- *Dieting*: Dutch Restraint Eating Scale (DRES) to assess frequency of dieting behaviors [31].
- *Binge eating*: will be measured by EDE-Q items 13, 14, 15 [32]

Mental health:

- *Anxiety symptoms*: Anxiety experienced within the past 14 days was measured using the Generalized Anxiety Disorder 2-item scale (GAD-2) [33].
- *Functional Impairment*: will be examined using the Clinical Impairment Assessment (CIA), to examine psychosocial impairment due to ED symptoms [34].
- *Depression symptoms*: Depression experienced within the past 14 days will be measured using the Patient Health Questionnaire 2-item scale (PHQ-2) [35].
- *Positive and Negative affect* will be assessed using *PANAS SF* [36].
- *Stress* will be assessed using the single item: “How often do you feel stressed?” [37].
- *Psychological distress* will be assessed using Brief Symptoms Inventory 18 (BSI-18) [38].
- Social Behavior will be assessed using *Social Adjustment Scale* [39].
- *Loneliness* will be assessed through BSI item 5.

Other

- *Demographic characteristics* will be collected, including familial and living status, work/educational status, somatic or psychiatric illness and related treatment, weight and height, physical activity, sleep problems, substance use, and eating disorders in family.
- *Health Care Utilization* during the past 1 month will be assessed using two items: (1) the number of times participants saw specific health care professionals because of physical or psychological illness, respectively, and (2) the number of times the participant saw a psychologist, psychiatrist, counselor, or therapist due to mental health problems.
- *Social media use* will be assessed using multiple items to generate two distinct measures: one capturing time spent on various platforms, and another evaluating the perceived influence of social media on body satisfaction and self-perception [40].

### Power calculations

The incidence of new onset of ED over a period of 2 years is determined via the EDDI at baseline and 1- and 2-year follow-up. Based on the incidence of ED in the study by Ghaderi and colleagues, we expect significant differences between groups, with an incidence of ED in the control group of 8.8% and 2% in the intervention group [16]. We accept a significance level of 0.05 (*α*) and a power of 0.80 (*β*), leading to a sample size of 137 in each arm. To accommodate for potential attrition, we aim to include 150 in each arm. Attrition rates have been reported as increasing by 12% at 2 years and 19% at 4-year follow-up [21].

### Statistical analysis

Analyses will follow the intention-to-treat (ITT) principle, with participants analyzed in their randomized groups. The primary outcome, onset of a clinical ED by 2-year follow-up, will be analyzed using logistic regression with study condition as the independent variable. Results will be reported as odds ratios (ORs) with 95% confidence intervals (CIs). Secondary outcomes, including continuous measures of ED symptoms, body dissatisfaction, psychiatric symptom load, and QoL across five timepoints (baseline, post-intervention, 6 months, 1 year, and 2 years), will be analyzed using linear mixed-effects models (LMMs). These models will include fixed effects for study condition, time, and their interaction, with random intercepts for participants and groups to account for within-subject correlation and clustering of participants within groups. Where appropriate, random slopes for time will be explored. Effect estimates will be reported with 95% CIs.

Baseline characteristics will be summarized using descriptive statistics. Between-group comparisons will use *t* tests or Mann–Whitney *U* tests for continuous variables (depending on distribution) and *χ*^2^ or Fisher’s exact tests for categorical variables. Effect modification will be examined by testing interactions between intervention assignment and baseline psychiatric diagnosis, as well as between intervention assignment and diagnostic complexity (single versus multiple psychiatric diagnoses), via interaction terms in the regression models. Missing data will be handled using multiple imputations. All statistical tests will be two-tailed with *α* = 0.05.

### Ethical considerations

The study conforms to the Declaration of Helsinki. The protocol has been approved by the Regional Committee on Health Research Ethics (Nr. 1–10–72–71–24) registered at the Danish data protection agency at Region Midtjylland (Nr. 1–16–02–92–25) and registered at ClinicalTrials.gov (NCT06893627).

Since some of the participants are under 18 years, all information related to the study has been tailored for this age group and has included representatives from this demographic in the testing process. Before including participants, information on the purpose, and potential risks or benefits of the study is given to all participants at the initial screening video call and afterward in written form to both them and their parents (if participants are between 15 and 17 years). All participants are provided a window of opportunity (24 h) to consider participation. If they choose to proceed, a written informed consent is obtained digitally from all participants via REDCap. Participants are notified that they are free to withdraw from the project at any time, without questions or consequences, and that they can request their data to be deleted. All data will be stored in a REDCap database at Aarhus University securing the General Data Protection Regulation.

With reference to the extensive previous Body Project literature, both including the virtual delivery mode and peer-led interventions, we consider Body Project to be safe and applicable for this project. We do not expect any adverse events in the SMILE Body Project trial as this has not previously been reported in similar settings; we will, however, continuously monitor participants on several parameters and register any adverse events, which will be discussed, and appropriate action will be taken. As this is an ED prevention program targeting individuals with body dissatisfaction, participants with a current ED diagnosis will be excluded from the study and advised on treatment options.

## Conclusions

This paper presents the study protocol for a randomized controlled trial evaluating the effectiveness of an ED prevention program for young women with psychiatric disorders, a group known to have a fourfold increased risk of developing EDs compared to their peers without psychiatric diagnoses. The co-existence of psychiatric illness and ED affects the outcome of both diseases, yet little is known about the prevention of ED behaviors and symptoms in this population. This study will extend previous international Body Project trials and contribute with knowledge of the effect on a specific high-risk group that has not been tested before.

The SMILE Body Project trial will provide a unique opportunity to investigate the efficacy of an online national prevention program for this vulnerable population. Should the SMILE Body Project prove effective, the results of this trial will have implications for the prevention of ED internationally?

With low cost and the promising results from prior studies, this could have a tremendous effect, both on the QoL in individuals with increased body dissatisfaction but also on national health care costs related to individuals with eating disorders.

## Study organization, dissemination policy, and status

The SMILE research group is located at Aarhus University Hospital Psychiatry. The Principal Investigator is last author LC, and the daily project manager is the first author CBA. The advisory board includes all authors and will meet twice yearly.

The findings from the SMILE Body Project trial will be disseminated to healthcare professionals, policymakers, and the broader public. Results will be reported in the trial registry and summarized in accessible, plain-language formats for participants and interested stakeholders. In addition, scientific findings will be communicated through presentations at conferences and publications in peer-reviewed journals.

The first participants were enrolled in the study in May 2025. Follow-up assessments for all participants are expected to be completed by November 2026.

## Supplementary Information

Below is the link to the electronic supplementary material.

Supplementary file1.

## Data Availability

No data sets were generated or analyzed during the current study.
