# Supplementary material for: SMILE body project: protocol for a randomized controlled trial of an online eating disorder prevention program in young women with psychiatric disorders
Source: Eat Weight Disord. 2026 Mar 11;31(1):37. doi: 10.1007/s40519-026-01832-w (PMC13095908; doi:10.1007/s40519-026-01832-w)
Supplement: Supplementary file 1 — Supplementary file1. [file 40519_2026_1832_MOESM1_ESM.docx]

**SPIRIT 2025 checklist of items to address in a randomized trial protocol***

| **Section / Topic** | **No** | **SPIRIT 2025 checklist item description** | **Reported on page no.** |
| --- | --- | --- | --- |
| **Administrative information** | | |  |
| Title and structured summary | 1a | Title stating the trial design, population, and interventions, with identification as a protocol | p. 1 : (Title: “SMILE Body Project: Protocol for a Randomized Controlled Trial of an Online Eating Disorder Prevention Program in Young Women with Psychiatric Disorders”) |
|  | 1b | Structured summary of trial design and methods, including items from the World Health Organization Trial Registration Data Set | p. 1 Abstract |
| Protocol version | 2 | Version date and identifier | ClinicalTrials.gov ID given (NCT06893627) – p.11 |
| Roles and responsibilities | 3a | Names, affiliations, and roles of protocol contributors | p.1 (Author list, affiliations) |
|  | 3b | Name and contact information for the trial sponsor | Corresponding author given (p.1) |
|  | 3c | Role of trial sponsor and funders in design, conduct, analysis, and reporting of trial; including any authority over these activities | p. 8 “Funding” |
|  | 3d | Composition, roles, and responsibilities of the coordinating site, steering committee, endpoint adjudication committee, data management team, and other individuals or groups overseeing the trial, if applicable | p. 8: “Study Organization, Dissemination policy and Status” |
| **Open science** | | |  |
| Trial registration | 4 | Name of trial registry, identifying number (with URL), and date of registration. If not yet registered, name of intended registry | \|  \| \| --- \|   ClinicalTrials.gov Identifier: NCT06893627   \| p.1 & p.10 (“ClinicalTrials.gov Identifier: NCT06893627”) \| \| --- \| |
| Protocol and statistical analysis plan | 5 | Where the trial protocol and statistical analysis plan can be accessed | Protocol published (this document), statistical plan in p.9 |
| Data sharing | 6 | Where and how the individual de-identified participant data (including data dictionary), statistical code, and any other materials will be accessible | A secure folder in MidtX at Aarhus University Hospital. |
| Funding and conflicts of interest | 7a | Sources of funding and other support (e.g., supply of drugs) | No other support or conflict of interest |
|  | 7b | Financial and other conflicts of interest for principal investigators and steering committee members | p.1 (“The authors have no relevant financial interests to disclose”) |
| Dissemination policy | 8 | Plans to communicate trial results to participants, healthcare professionals, the public, and other relevant groups (e.g., reporting in trial registry, plain language summary, publication) | p. 8: “Study Organization, Dissemination policy and Status” |
| **Introduction** | | |  |
| Background and rationale | 9a | Scientific background and rationale, including summary of relevant studies (published and unpublished) examining benefits and harms for each intervention | p.2–4 (ED prevalence, risks in psychiatric illness, Body Project literature) |
|  | 9b | Explanation for choice of comparator | p.6 (Active control = expressive writing, credible alternative used in prior trials) |
| Objectives | 10 | Specific objectives related to benefits and harms | p.4–5 (Hypotheses I–IV) |
| **Methods: Patient and public involvement, trial design** | | |  |
| Patient and public involvement | 11 | Details of, or plans for, patient or public involvement in the design, conduct, and reporting of the trial | p.10 (testing study info with <18 group; tailoring materials) |
| Trial design | 12 | Description of trial design including type of trial (e.g., parallel group, crossover), allocation ratio, and framework (e.g., superiority, equivalence, non-inferiority, exploratory) | p.4 (randomized controlled trial, 1:1 allocation, superiority design) |
| **Methods: Participants, interventions, and outcomes** | | |  |
| Trial setting | 13 | Settings (e.g., community, hospital) and locations (e.g., countries, sites) where the trial will be conducted | p.5 (Recruitment via psychiatry departments, schools, social media, Central & Northern Denmark Regions) |
| Eligibility criteria | 14a | Eligibility criteria for participants | p.5–6 (Table 1; inclusion & exclusion) |
|  | 14b | If applicable, eligibility criteria for sites and for individuals who will deliver the interventions (e.g., surgeons, physiotherapists) | p.6–7 (Facilitators = trained peers with psychiatric experience, healthcare students) |
| Intervention and comparator | 15a | Intervention and comparator with sufficient details to allow replication including how, when, and by whom they will be administered. If relevant, where additional materials describing the intervention and comparator (e.g., intervention manual) can be accessed | p.6–7 (SMILE Body Project: 4 online sessions, peer-led; comparator = expressive writing; Table 2 content) |
|  | 15b | Criteria for discontinuing or modifying allocated intervention/comparator for a trial participant (e.g., drug dose change in response to harms, participant request, or improving/worsening disease) | Participants may withdraw anytime (p.10) |
|  | 15c | Strategies to improve adherence to intervention/comparator protocols, if applicable, and any procedures for monitoring adherence (e.g., drug tablet return, sessions attended) | \|  \| p.6 (recorded sessions, fidelity checks, follow-up  phone calls for missed sessions) \| \| --- \| --- \| |
|  | 15d | Concomitant care that is permitted or prohibited during the trial | p.4 (all participants continue usual psychiatric care; no restrictions) |
| Outcomes | 16 | Primary and secondary outcomes, including the specific measurement variable (e.g., systolic blood pressure), analysis metric (e.g., change from baseline, final value, time to event), method of aggregation (e.g., median, proportion), and time point for each outcome | p.7–8 (Primary: ED diagnosis via EDDI; Secondary: body dissatisfaction, thin-ideal, psychiatric symptoms, QoL, etc.) |
| Harms | 17 | How harms are defined and will be assessed (e.g., systematically, non-systematically) | p.10 (continuous monitoring, adverse events registered, no expected harms) |
| Participant timeline | 18 | Time schedule of enrollment, interventions (including any run-ins and washouts), assessments, and visits for participants. A schematic diagram is highly recommended (see Figure) | p.4 (flowchart Figure 1), p.7–9 (baseline, post, 6mo, 1y, 2y) |
| Sample size | 19 | How sample size was determined, including all assumptions supporting the sample size calculation | p.9 (power calc: 300 participants, 150 per arm, based on incidence differences) |
| Recruitment | 20 | Strategies for achieving adequate participant enrollment to reach target sample size | p.5 (leaflets, SoMe ads, website, psychiatric departments, schools) |
| **Methods: Assignment of interventions** | | |  |
| Randomization: |  |  |  |
| Sequence generation | 21a | Who will generate the random allocation sequence and the method used | p.4 (block randomization with varying block sizes; details of allocation concealment, created by REDcap admin. Jacob Hjort, Aarhus University) |
|  | 21b | Type of randomization (simple or restricted) and details of any factors for stratification. To reduce predictability of a random sequence, other details of any planned restriction (e.g., blocking) should be provided in a separate document that is unavailable to those who enroll participants or assign interventions | p.4 (random permutations within block to ensure concealment) |
| Allocation concealment  mechanism | 22 | Mechanism used to implement the random allocation sequence (e.g., central computer/telephone; sequentially numbered, opaque, sealed containers), describing any steps to conceal the sequence until interventions are assigned | p.4 (block randomization with varying block sizes; details of allocation concealment, created by REDcap admin. Jacob Hjort, Aarhus University) |
| Implementation | 23 | Whether the personnel who will enroll and those who will assign participants to the interventions will have access to the random allocation sequence | No |
| Blinding | 24a | Who will be blinded after assignment to interventions (e.g., participants, care providers, outcome assessors, data analysts) | Not blinded (open-label RCT); p.4–5 implies participants and facilitators know assignment |
|  | 24b | If blinded, how blinding will be achieved and description of the similarity of interventions | N.A |
|  | 24c | If blinded, circumstances under which unblinding is permissible, and procedure for revealing a participant’s allocated intervention during the trial | N.A |
| **Methods: Data collection, management, and analysis** | | |  |
| Data collection methods | 25a | Plans for assessment and collection of trial data, including any related processes to promote data quality (e.g., duplicate measurements, training of assessors) and a description of trial instruments (e.g., questionnaires, laboratory tests) along with their reliability and validity, if known. Reference to where data collection forms can be accessed, if not in the protocol | p.7–8 (EDDI, validated questionnaires; translated/back-translated; REDCap use) |
|  | 25b | Plans to promote participant retention and complete follow-up, including list of any outcome data to be collected for participants who discontinue or deviate from intervention protocols | p.6 (follow-up phone if sessions missed), p.7–9 (multiple follow-ups over 2 years) |
| Data management | 26 | Plans for data entry, coding, security, and storage, including any related processes to promote data quality (e.g., double data entry; range checks for data values). Reference to where details of data management procedures can be accessed, if not in the protocol | p.10 (all data stored in REDCap at Aarhus University; GDPR compliant) |
| Statistical methods | 27a | Statistical methods used to compare groups for primary and secondary outcomes, including harms | p.9 (logistic regression for primary; linear mixed models for secondary) |
|  | 27b | Definition of who will be included in each analysis (e.g., all randomized participants), and in which group | p.9 (intention-to-treat principle) |
|  | 27c | How missing data will be handled in the analysis | Missing data will be handled using multiple imputation, p. 7. |
|  | 27d | Methods for any additional analyses (e.g., subgroup and sensitivity analyses) | p.9 (exploratory analyses: moderators like psychiatric symptoms, social media use, etc.) |
| **Methods: Monitoring** | | |  |
| Data monitoring committee | 28a | Composition of data monitoring committee (DMC); summary of its role and reporting structure; statement of whether it is independent from the sponsor and funder; conflicts of interest and reference to where further details about its charter can be found, if not in the protocol. Alternatively, an explanation of why a DMC is not needed | The data monitoring committee is the research group who is independent of sponsors. p. 8: “Study Organization, Dissemination policy and Status” |
|  | 28b | Explanation of any interim analyses and stopping guidelines, including who will have access to these interim results and make the final decision to terminate the trial | We plan to adjust the control arm to no intervention if > 5 participant report adverse events of the expressive writing condition. |
| Trial monitoring | 29 | Frequency and procedures for monitoring trial conduct. If there is no monitoring, give explanation | p.6–7 (fidelity checks, by-monthly supervision for facilitators) but no formal monitoring committee |
| **Ethics** | | |  |
| Research ethics approval | 30 | Plans for seeking research ethics committee/institutional review board approval | p.10 (approved by Regional Committee on Health Research Ethics; registered with data protection agency) |
| Protocol amendments | 31 | Plans for communicating important protocol modifications to relevant parties | Adjustments will be reported for approval at the local Ethics department. |
| Consent or assent | 32a | Who will obtain informed consent or assent from potential trial participants or authorized proxies, and how | p.10 (information at screening call, written consent via REDCap; assent for <18 with parental info) |
|  | 32b | Additional consent provisions for collection and use of participant data and biological specimens in ancillary studies, if applicable | NA |
| Confidentiality | 33 | How personal information about potential and enrolled participants will be collected, shared, and maintained in order to protect confidentiality before, during, and after the trial | p.10 (GDPR, REDCap secure storage, withdrawal option with data deletion) |
| Ancillary and post-trial care | 34 | Provisions, if any, for ancillary and post-trial care, and for compensation to those who suffer harm from trial participation | p.10–11 (participants with current ED excluded & referred for treatment; otherwise no ancillary/post-trial care mentioned) |

*We strongly recommend reading this checklist in conjunction with the SPIRIT 2025 Explanation and Elaboration and the SPIRIT 2025 Expanded Checklist for important clarifications on all the items. We also recommend reading relevant SPIRIT extensions. See [www.consort-spirit.org](http://www.consort-spirit.org)

Citation: Chan A-W, Boutron I, Hopewell S, Moher D, Schulz KF, et al. SPIRIT 2025 statement: updated guideline for protocols of randomised trials. BMJ 2025;389:e081477. <https://dx.doi.org/10.1136/bmj-2024-081477>

© 2025 Chan A-W et al. This is an Open Access article distributed under the terms of the Creative Commons Attribution License (<https://creativecommons.org/licenses/by/4.0/>), which permits unrestricted use, distribution, and reproduction in any medium, provided the original work is properly cited.
